# Dielectric Modes in Antiferroelectric and Ferroelectric Liquid Crystals in a Pure Enantiomeric Version and a Racemic Mixture

**DOI:** 10.3390/ma17133335

**Published:** 2024-07-05

**Authors:** Paweł Perkowski, Magdalena Urbańska

**Affiliations:** 1 Institute of Applied Physics, Military University of Technology, Kaliskiego 2, 00-908 Warsaw, Poland; 2 Institute of Chemistry, Military University of Technology, Kaliskiego 2, 00-908 Warsaw, Poland; magdalena.urbanska@wat.edu.pl

**Keywords:** antiferroelectric liquid crystal, ferroelectric liquid crystal, (S) enantiomer, racemate, chirality, dielectric spectroscopy, relaxation mode, activation energy, Arrhenius law

## Abstract

The dielectric properties of synclinic (ferroelectric SmC*) and anticlinic (antiferroelectric SmC_A_*) smectic liquid crystals composed of molecules of one chiral version (S) are presented and compared with properties of racemic mixture (R, S), showing SmC and SmC_A_ phases. The racemic mixture completely loses its ferroelectric and antiferroelectric properties. Surprisingly, only one dielectric mode observed in the antiferroelectric SmC_A_* phase disappeared in the dielectric response of the racemic SmC_A_ phase. Additionally, we observed that in the SmC phase, seen in the racemic mixture, the weak dielectric mode (named the X mode) is detected, which seems to be the continuation of the P_L_ mode existing in the racemic SmC_A_. Moreover, this mode in the racemic SmC has nothing to do with the Goldstone mode, typical for the SmC* phase. This paper describes in detail the real and imaginary parts of dielectric permittivity in smectic phases for the enantiomer and racemate with and without a DC field, compares the properties of the X and P_L_ modes, and discusses the full scheme of dielectric modes in enantiomer and racemate.

## 1. Introduction

The definition of chirality was coined by William Thomson (Lord Kelvin) in his “Robert Boyle Lecture” at Oxford University in 1893. He wrote the following in his “*Baltimore Lectures on Molecular Dynamics and the Wave Theory of Light*” in 1904 [1]: “I call any geometrical figure or group of points chiral and say it has chirality, if its image in a plane mirror, ideally realized, cannot be brought to coincide with itself. Two equal and similar right hands are homochirally similar. Equal and similar right and left hands are heterochirally similar”.

Chirality is an important factor in life. Indeed, all amino acids (except glycine) that build the proteins in our bodies are chiral molecules. Moreover, natural amino acids appear only in one chiral form. Our nose can recognize the chirality of heterochirally similar natural substances. Additionally, medicine in one chiral form can cure while the opposite form of the same medicine can be poisonous.

In general, chirality plays an important role in organic chemistry. Chirality is the main feature of molecules building liquid crystals, which leads to the polar properties of ferro-, antiferro-, and ferrielectricity in mesophases and the creation of helical superstructures. The source of polar properties differs from “proper” polar phases. In the “proper” polar phases, spontaneous polarization results from the effect of a high density of strong dipole moments that interact electrically with each other [2,3,4], as is observed in nematic ferroelectric liquid crystals. In the “improper” polar phases, spontaneous polarization in tilted smectic phases results from chirality and mirror symmetry breaking [5,6]. Chirality introduced into the molecular structure makes the rotational potential around the long molecular axis asymmetric [7,8,9,10]. Hence, spontaneous polarization appears in the direction perpendicular to the tilt plane in the SmC* and SmC_A_* phases. After the discovery of liquid crystals by Reinitzer in 1888 [11], it took 90 years to discover improper ferroelectricity in 1978 in DOBAMC [12]. It took another 10 years until improper antiferroelectricity was found in liquid crystals [13]. Additionally, another 30 years passed before proper ferroelectricity was found in nematics in 2016 [2,3,4,14].

The dielectric response of the polar phase strongly differs from the dielectric response of the racemic version of the investigated liquid crystal. The best example is the strong Goldstone mode visible in the ferroelectric SmC* phase [15,16]. In the racemic analog of the SmC* phase (racemic SmC), this strong mode is not detectable [17,18,19] because of the disappearance of the helical superstructure.

The antiferroelectric SmC_A_* phase exhibits two collective modes, P_H_ and P_L_, at room temperature, as described in many papers [20,21,22,23,24,25,26]. These are called non-cancelled modes because they exist only for non-perfect anticlinic double-layer structures. The director in the first layer is not exactly on the opposite side of the cone, in comparison with the director in the next smectic layer, because of the small rotation caused by the helical superstructure. Hence, net polarization can exist in the double-layer unit. In a perfect double-layer structure, both modes would be canceled and non-active in dielectric response [15]. The P_H_ mode is faster and is called the anti-phase phason mode. For this collective mode, molecules in neighboring layers rotate (on the cone) in opposite directions. The P_L_ mode is slower and is called the in-phase phason mode. For this collective mode, molecules in neighboring layers rotate (on the cone) in the same direction. Additionally, one molecular mode called the S mode (molecular rotation around the short molecular axis), faster than the P_H_ mode, is also visible at room temperature, while the molecular L mode (molecular rotation around the long molecular axis) is too fast to be detectable at room temperature using standard impedance analyzers. When the antiferroelectric phase nucleates from the ferroelectric phase at cooling, one can notice that the amplitude of the Goldstone mode in the SmC* phase is dozens of times stronger than those of modes P_H_ and P_L_ [27]. As mentioned above, this results from the fact that the polarizations from adjacent layers in the SmC_A_* phase are close to being canceled, while in the SmC* phase, they add up.

The DC field is often used to investigate electrically active modes. It can modify the dielectric response of ferroelectric and antiferroelectric phases. The DC field suppresses the Goldstone mode in the SmC* phase because it blocks the molecular movement on the cone by the unwinding of the helix [28]. In the SmC_A_* phase, the DC influence is a little different. Two modes (P_H_ and P_L_) are gradually strengthened by increasing the low DC field, while the S mode is weakened. When the DC field is high enough, both collective modes disappear because of the cancellation of the helical superstructure. For the suppression of both the P_H_ and P_L_ modes, we need a higher DC field than for the suppression of the Goldstone mode. Hence, when we suppress (using the DC field) the Goldstone mode at the SmC*-SmC_A_* phase transition, we can see both antiferroelectric modes, which are normally hidden by the ferroelectric mode [29,30].

Another way to suppress the Goldstone mode as a sign of ferroelectricity is via racemization. When we use this method, we can see the dielectric response usually hidden by strong Goldstone mode. Earlier investigations [17,18,19] show that the dielectric response of a racemic mixture is richer than we can expect, and some modes in racemate behave as modes in an enantiomer.

This paper’s main goal is to answer the question of how racemization influences the dielectric response of anticlinic SmC_A_ and synclinic SmC phases in comparison with the dielectric response of enantiomer and its anticlinic SmC_A_* and synclinic SmC* phases.

The symbols used in this paper include the following:

SmC* —ferroelectric synclinic phase with a helical superstructure and its axis of rotation perpendicular to the smectic layers. Molecules in one smectic layer are tilted by the angle θ.
SmC_A_* —antiferroelectric synclinic phase with a helical superstructure and its axis of rotation perpendicular to the smectic layers. Molecules in one smectic layer are tilted by the angle θ, while molecules in the next layer are tilted by the angle −θ.
SmC —synclinic phase without a helical superstructure. Molecules in one smectic layer are tilted by the angle θ. The helical superstructure and ferroelectricity vanish when the SmC phase is built from achiral molecules or SmC is the racemate—the phase is a mixture of 50% S-enantiomer and 50% R-enantiomer.
SmC_A_ —anticlinic phase without a helical superstructure. Molecules in one smectic layer are tilted by the angle θ, while molecules in the next layer are tilted by the angle −θ. The helical superstructure and antiferroelectricity vanish when the SmC_A_ phase is built from achiral molecules or SmC_A_ is the racemate—the phase is an equimolar mixture of S- and R-enantiomers.
Goldstone mode —collective and strong dielectric mode (molecules move collectively around the cone in the smectic layer) in the same direction. During the rotation the phase angle changes in time, so this mode is called the phason mode.
S mode —molecular dielectric mode when the molecule rotates around its short molecular axis. This mode is observed in isotropic liquid, nematic, or smectic phases. This mode is Arrhenius type: its relaxation frequency decreases with decreasing temperature. The relaxation frequency of the S mode is 1k–10k times lower than the relaxation frequency of the L mode.
L mode —molecular dielectric mode when the molecule rotates around its long molecular axis. This mode is observed in isotropic liquid, nematic, or smectic phases. This mode is also Arrhenius type. It is not usually observed in standard impedance spectroscopy because it is fast.
P_H_ mode —collective and weak (in comparison with the Goldstone mode) dielectric mode detectable in antiferroelectric anticlinic SmC_A_*. It is also Arrhenius type. Molecules in neighboring layers, in the two-layer unit, rotate in opposite directions around the cone; hence, it is called the anti-phase phason mode. The relaxation frequency of the P_H_ mode is 1k times lower than the relaxation frequency of the S mode and 1k times higher than the relaxation frequency of the P_L_ mode.
P_L_ mode —collective and weak (in comparison with Goldstone mode) dielectric mode detectable in antiferroelectric anticlinic SmC_A_*. It is also Arrhenius type. Molecules in neighboring layers, in the two-layer unit, rotate in the same direction around the cone; hence, it is called the in-phase phason mode. The relaxation frequency of the P_L_ mode is 1k lower than the relaxation frequency of the P_H_ mode.
X mode —the dielectric mode described in this paper, which is observed in the racemic synclinic SmC phase. It seems to be the continuation of the P_L_ mode from the racemic SmC_A_ phase. Its strength at the SmC-SmC_A_ phase transition is twice weaker than the strength of the P_L_ mode close to the SmC_A_-SmC phase transition.
$\delta \varepsilon_{i}$ —the strength of the *i*th dielectric mode.
$f_{ri}$ —the relaxation frequency of the *i*th dielectric mode.
$\varepsilon_{\infty }$ —high-frequency limit of permittivity.
$\varepsilon_{S}$ —low-frequency limit of permittivity.
$\alpha_{i}$ and $\beta_{i}$ —Havriliak–Negami distribution parameters.
j —imaginary unit.
$\sigma_{ion}$ —ionic conductivity of the sample.
n —parameter describing ion contribution to the imaginary part of permittivity (close to one).
C —capacity in the parallel equivalent circuit.
G —conductivity in the parallel equivalent circuit.
$C_{0}$ —empty cell capacity.
$f_{\infty }$ —limit of relaxation frequency at high temperatures (in Arrhenius law).
k —Boltzmann constant.
T —temperature on the thermodynamic scale.

## 2. Materials and Methods

### 2.1. Studied Compound

**Figure 1:**
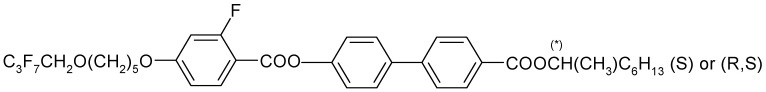
The molecular structure of the investigated liquid crystal. The asterisk (*) indicates where the chiral carbon atom (center) in the molecular structure is.

To show how racemization influences the dielectric response of the antiferroelectric SmC_A_* and ferroelectric SmC* phases, the monofluorinated compound [31] and its racemic mixture [32] (Figure 1) were studied using dielectric spectroscopy. Importantly, the pure chiral compound (S) and its racemic mixture (R, S) exhibit both the anticlinic and synclinic phases.

### 2.2. DSC Measurements

The transition temperatures and enthalpies of the transition for the enantiomer and racemate were determined by differential scanning calorimetry (DSC) using a SETARAM 141 microcalorimeter (KEP Technologies, Caluire-Et-Cuire, France) at the heating/cooling rate of 2 °C/min. The weight of each sample was about 20–30 mg.

### 2.3. Microscopy Observations

Mesophases were identified by observing the textures using the OLYMPUS BX51 optical microscope (Olympus Co., Tokyo, Japan) under crossed polarizers with the Linkam THMS-600 hot stage (Linkam Scientific Instruments LTD, Salfords, UK) controlled by the Linkam TMS-93 temperature programmer at the heating/cooling rate of 2 or 3 °C/min.

### 2.4. Dielectric Spectroscopy

Impedance spectroscopy is a useful method to characterize the dielectric properties of liquid crystals. The HP 4192A impedance analyzer (Hewlett Packard Inc., Palo Alto, CA, USA) was used in our investigations. This equipment allows, nominally, to take measurements at frequencies from 5 Hz to 13 MHz. Our previous experience with liquid crystal research led us to conclude that the measurement range (with acceptable accuracy) is narrower from 100 Hz to 10 MHz. At lower frequencies, ions distort the measurements, while at higher frequencies, the measurements are influenced by well-known high-frequency parasitic effects [33]. Our impedance analyzer enables applying the DC field from 0 V to 30 V, and it does not influence the measuring procedure at different AC signals. Hence, the DC field influence on the dielectric properties of the measured medium can be easily observed. In our investigation, the sign of the DC field was not important.

For measurements, self-made cells with gold electrodes (with an area of 25 mm^2^), to minimize high-frequency parasitic effects, were used. Such cells can be used for frequencies up to 10 MHz. The thickness of the used cells was around 5 µm, while the cell’s alignment was planar. The aligning layer was polyimide SE130 (Nissan Chemical Corporation, Tokyo, Japan). Liquid crystals were heated and put in a measuring cell in the isotropic phase using capillary action. Six measuring cycles were performed to understand the dielectric properties fully. The measurements were performed without a DC field and under 5 V or 10 V DC fields for the racemic mixture and pure enantiomer. In this paper, the results obtained at the 5 V DC field are not presented. All measurements were performed on the cooling cycles (starting from isotropic liquid). To control the temperature, the Linkam THMS-600 hot stage (Linkam Scientific Instruments LTD, Salfords, UK) controlled by a Linkam TMS-92 was used. The cooling rate was 0.5 °C/min.

The measurements were performed in a parallel equivalent circuit as follows: capacity C together with the conductivity G. Knowing the circuit parameters versus frequency $C\left(f\right)$ and $G\left(f\right)$, the real $\varepsilon^{\prime }$ and imaginary $\varepsilon^{\prime \prime }$ parts of the permittivity of the investigated compounds can be calculated using simple formulas as follows: $\varepsilon^{\prime }\left(f\right)=C\left(f\right)/C_{0}$ and $\varepsilon^{\prime \prime }\left(f\right)=G\left(f\right)/2\pi fC_{0}$, where $C_{0}$ stands for empty cell capacity. $C_{0}$ was measured in an experimental setup just before filing the cell with liquid crystal.

Usually, the pure results of the dielectric spectroscopy of liquid crystals obtained in experiments are presented in the following four ways: (1) the real part $\varepsilon^{\prime }$ of the permittivity plot (at several frequencies) vs. temperature, (2) the imaginary part $\varepsilon^{\prime \prime }$ of the permittivity plot (at several frequencies) vs. temperature, (3) the real part $\varepsilon^{\prime }$ of the permittivity plot (at several temperatures) vs. frequency (frequency spectrum), and (4) the imaginary part $\varepsilon^{\prime \prime }$ of the permittivity plot (at several temperatures) vs. frequency (frequency spectrum). The first and second ways are preferred when the confirmation of existing liquid crystal phases is important. The third and fourth ways are suitable when the dielectric properties of each phase are analyzed. In this paper, we use the first and third methods of presentation.

### 2.5. The Method for Calculating the Parameters of Dielectric Modes

The main problem related to the dielectric spectroscopy of liquid crystals is knowing the true permittivity of liquid crystals. Parasitic effects make the permittivity measured in the experiment not exactly equal to the permittivity of liquid crystal [34]. This means that true values of liquid crystal permittivity should be calculated from experimental values. Fortunately, when cells with gold electrodes are used in measurements. the difference between the permittivity measured in the experiment and the true permittivity of a liquid crystal is small. The whole numerical procedure used in our calculations is explained in [35]. Important parameters in this procedure are the cut-off frequency of the RC measuring circuit and the resonance frequency of the LC measuring circuit.

Knowing the true permittivity of a given liquid crystal, one can calculate the parameters of the dielectric modes observed in an experiment. For the calculation of complex liquid crystal permittivity, Formula (1) was used (the Havriliak–Negami model [36]).

(1) $$\varepsilon^{*}=\varepsilon^{\prime }-j\varepsilon^{\prime \prime }=\varepsilon_{\infty }+\sum_{i=1}^{3}\frac{\delta \varepsilon_{i}}{{\left[1+{\left(jf/f_{ri}\right)}^{1-\alpha_{i}}\right]}^{\beta_{i}}}-j\frac{\sigma_{ion}}{{\left(2\pi f\right)}^{n}C_{0}},$$

where $\varepsilon_{\infty }$ stands for the high-frequency limit of permittivity, $\delta \varepsilon_{i}$ stands for the strength of *i*th mode, $f_{ri}$ stands for relaxation frequency of *i*th mode, $\alpha_{i}$ and $\beta_{i}$ stand for the Havriliak–Negami distribution parameters, j stands for an imaginary unit, $\sigma_{ion}$ stands for the ionic conductivity of the sample, n stands for a parameter (close to one), and $C_{0}$ stands for empty cell capacity. Calculations were performed for different phases. The maximum number i of the considered modes was 3.

The most important parameters found using Equation (1) are the relaxation frequencies $f_{ri}$ and strengths $\delta \varepsilon_{i}$ of the analyzed modes. Knowing the relaxation frequencies of analyzed modes vs. temperature, the activation energy $E_{a}$ from the Arrhenius law (2) can be calculated [37]. Of course, the relaxation frequency of the analyzed mode should follow the Arrhenius law.

(2) $$f_{r}\left(T\right)=f_{\infty }\exp\left(-\frac{E_{a}}{kT}\right),$$

where $f_{r}$ stands for relaxation frequency, $f_{\infty }$ stands for the limit of relaxation frequency at high temperatures, k stands for Boltzmann constant, and T stands for the temperature on the thermodynamic scale.

## 3. Results

### 3.1. Results of the DSC Measurements and Microscopic Observations Performed for the Pure Enantiomer and Racemate

From DSC and polarizing optical microscopy, one can find that the pure enantiomer (in both the S and R forms) exhibits the following phase transition scheme: Cr 28.1 °C SmC_A_* 99.0 °C SmC* 100.2 °C SmA* 101.1 °C Iso. Meanwhile, the racemic mixture manifests a slightly different phase sequence: Cr 39.1 °C SmC_A_ 84.3 °C SmC 95.1 °C Iso. The DSC plots for racemate and enantiomer are presented in the Supplementary Materials. 

**Figure 2:**
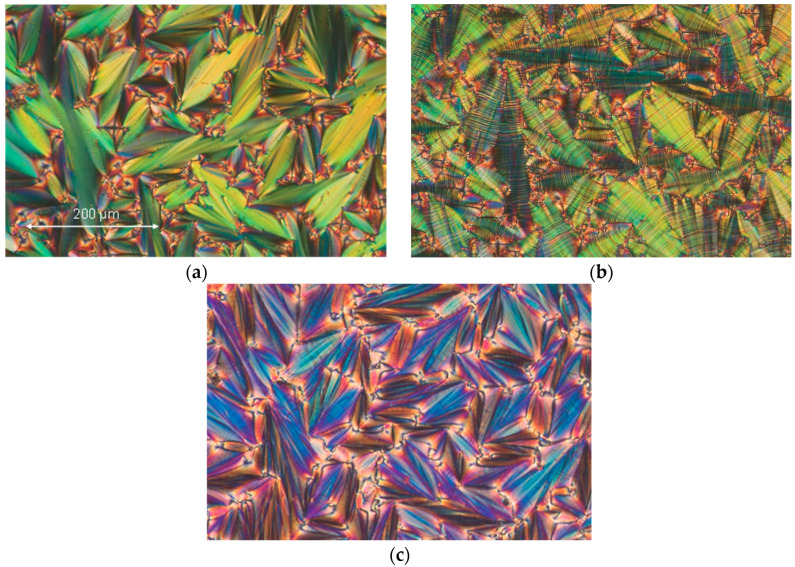
Photos showing the microscopic pattern of the racemate taken during the cooling cycle: (**a**) the SmA* phase at 98.5 °C, (**b**) the SmC* phase at 98.1 °C, and (**c**) the SmC_A_* phase at 88.0 °C.

**Figure 3:**
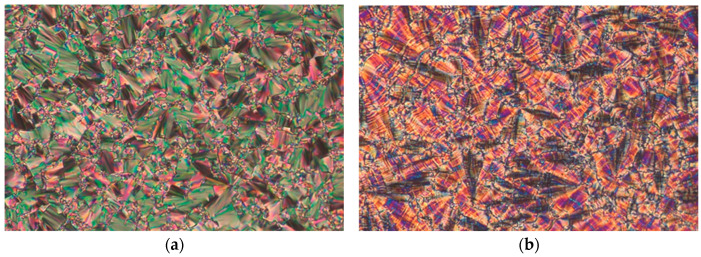
Photos showing the microscopic pattern of the racemate taken during the cooling cycle: (**a**) the SmC phase at 94.7 °C and (**b**) the SmC_A_ phase at 76.8 °C.

The textures of liquid crystalline phases for the enantiomer and the racemate are also different, as shown in Figure 2 and Figure 3. The width of all the microphotographs is about 600 μm. In the enantiomeric version, textures in both the SmC* and SmC_A_* phases manifest clear dechiralization lines (Figure 2b,c) because of the existence of a helical superstructure. When the racemate is observed, the SmC phase shows no dechiralization lines (Figure 3a). Surprisingly, in the SmC_A_ phase (Figure 3b), one can see weak and fuzzy dechiralization lines. This suggests that the compensation in the prepared racemate is not full.

### 3.2. Results of the Measurements Performed for the Pure Enantiomer

**Figure 4:**
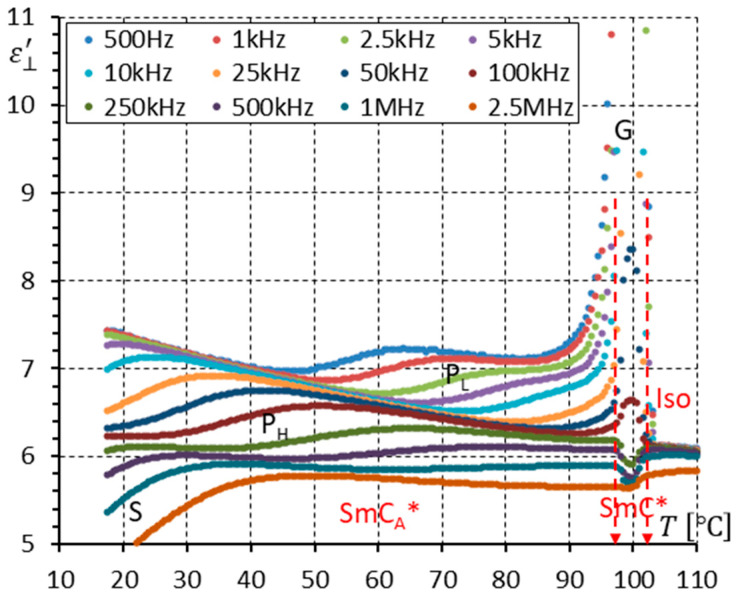
The real part $\varepsilon_{⊥}^{\prime }$ of permittivity for twelve measuring frequencies versus temperature T for the enantiomer (no DC field applied). The vertical scale allows us to see the permittivity in the SmC* phase (G—the Goldstone mode) and the permittivity in the SmC_A_* phase (three modes (P_L_, P_H_, S)) simultaneously. Arrows indicate phase transitions.

Figure 4 shows the real part $\varepsilon_{⊥}^{\prime }$ of permittivity (measured in a planarly aligned cell) versus temperature for twelve frequencies. This way of presenting dielectric properties is convenient for showing how the dielectric response changes when different phases appear with temperature. It is obvious when the dielectric properties exhibit dispersion. One can see poor dispersion in isotropic liquid and a strong dielectric mode, known as the Goldstone mode (G), seen in the SmC* phase (ferroelectric phason mode). The maximum permittivity value in the SmC* is around 170 (at 100 °C). The mesophase below the SmC* exhibits lower permittivity (the permittivity is comparable to isotropic liquid’s permittivity: ~6). Three modes are seen in this phase. They are the P_L_, P_H_, and S modes in the SmC_A_* phase. The first mode, P_L_, is called the in-phase antiferroelectric phason mode, the second mode, P_H_, is called the anti-phase antiferroelectric phason mode, and the last S mode is called the molecular mode around the short molecular axis. Both phases, SmC* and SmC_A_*, seem to behave classically, as described in the literature [18,20,21,23,24].

**Figure 5:**
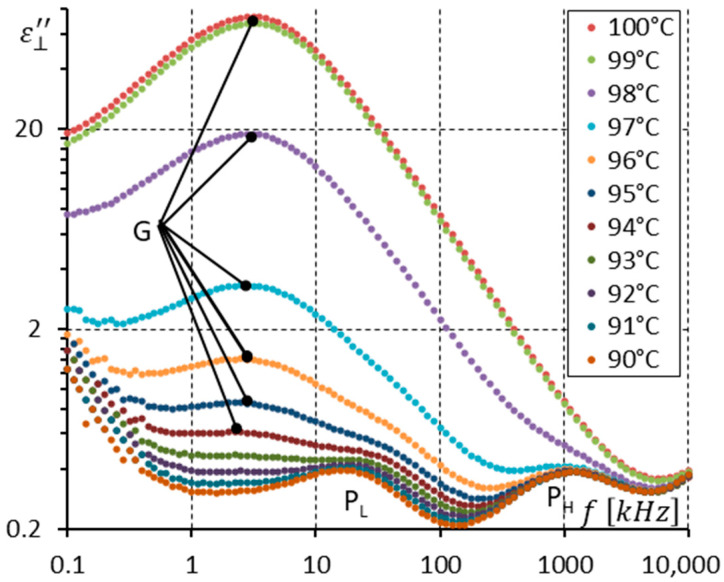
The imaginary part $\varepsilon_{⊥}^{\prime \prime }$ of permittivity for eleven temperatures (in the SmC* and SmC_A_* phases, close to the SmC*-SmC_A_* phase transition) versus measuring frequency f for the enantiomer (no DC field applied).

**Figure 6:**
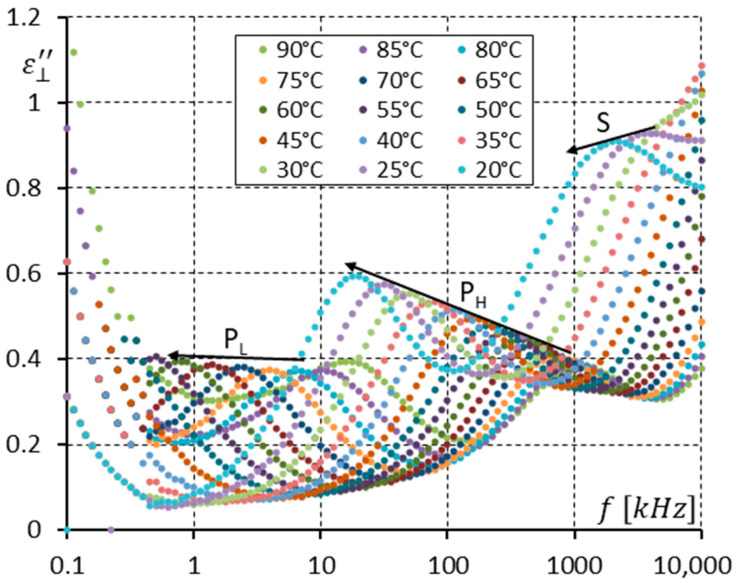
The imaginary part $\varepsilon_{⊥}^{\prime \prime }$ of permittivity for fifteen temperatures (in the SmC_A_* phase only) versus measuring frequency f for the enantiomer (no DC field applied). Three relaxations are seen. Arrows show how the maximum amplitude of $\varepsilon_{⊥}^{\prime \prime }$ (also the relaxation frequencies) shifts when the temperature decreases.

Figure 5 and Figure 6 show the imaginary part $\varepsilon_{⊥}^{\prime \prime }$ of permittivity versus frequency for several temperatures in both the SmC* and SmC_A_* phases (Figure 5) and in SmC_A_* only (Figure 6). In Figure 5, the Goldstone mode (G) gradually disappears with decreasing temperature, while both the P_L_ and P_H_ modes gradually appear. Alternatively, it can be said that the weak P_L_ and P_H_ modes that exist in the dielectric response of the SmC* phase (close to the SmC*-SmC_A_* phase transition) become gradually visible. We see that the Goldstone mode and the P_L_ and P_H_ modes coexist in the 97–91 °C temperature range. It is difficult to indicate precisely the transition temperature SmC*-SmC_A_*. This phase transition (second rank) is a continuous process.

In Figure 6, only the spectra for the antiferroelectric (SmC_A_*) phase are shown. Three modes are seen with their relaxation frequencies well separated in the frequency domain. When the temperature decreases, all relaxation frequencies also decrease (see arrows in Figure 6). At room temperature (25 °C), the S mode relaxation frequency is around 3.5 MHz, the P_H_ mode is simultaneously around 28 kHz, while the P_L_ mode is below 100 Hz. All three modes are Arrhenius-like. The P_H_ mode becomes stronger when the temperature decreases. The same effect should be also seen for the P_L_ and S modes. It is not visible because of the ion contribution (for the P_L_ mode) and high-frequency parasitic effects (for the S mode) [33]. Owing to the ion contribution, any existing modes at frequencies below the relaxation frequency of the P_L_ mode can not be seen.

**Figure 7:**
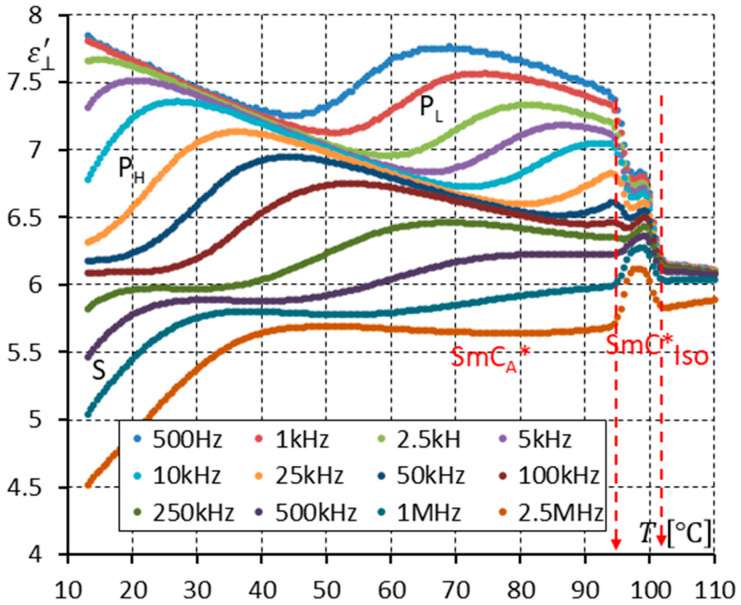
The real part $\varepsilon_{⊥}^{\prime }$ of permittivity for twelve measuring frequencies versus temperature T for the enantiomer (10 V DC field applied). Three modes (P_L_, P_H_, S) are seen in the SmC_A_* phase, while the Goldstone mode is fully suppressed in the SmC* phase. Arrows indicate the phase transitions.

**Figure 8:**
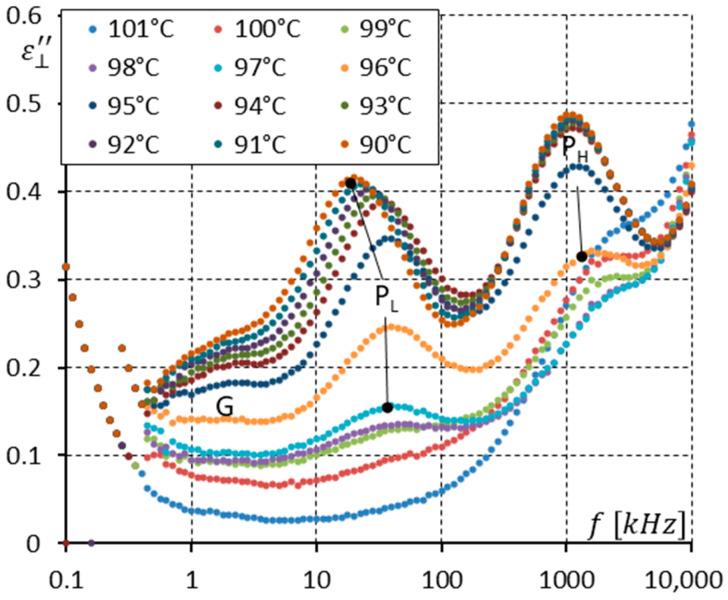
The imaginary part $\varepsilon_{⊥}^{\prime \prime }$ of permittivity for twelve temperatures (in the SmC* and the SmC_A_* phases) versus measuring frequency f for the enantiomer (10 V DC field applied).

**Figure 9:**
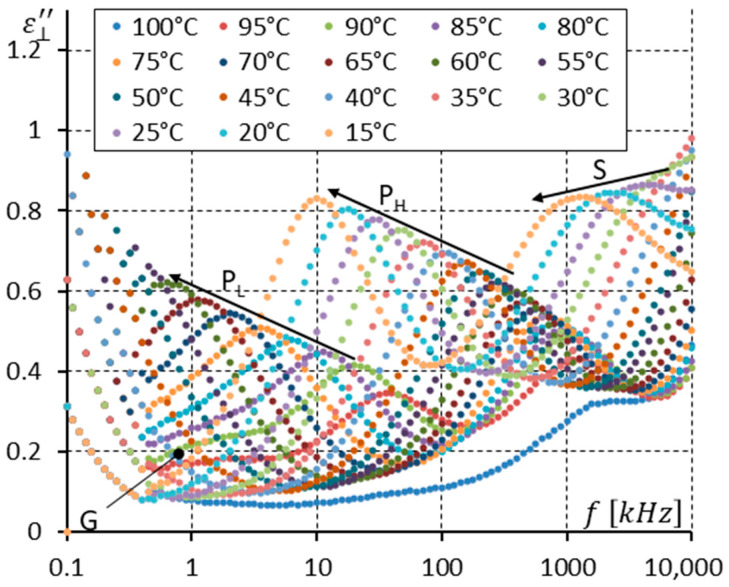
The imaginary part $\varepsilon_{⊥}^{\prime \prime }$ of permittivity for eighteen temperatures (in the SmC_A_* phase and one temperature (100 °C) in the SmC* phase) versus measuring frequency f for the enantiomer (10 V DC field applied). Arrows show how the maximum amplitude of $\varepsilon_{⊥}^{\prime \prime }$ (also the relaxation frequencies) shifts when the temperature decreases. The residual Goldstone mode (G) is also seen.

After applying the DC field (10 V) during measurement (Figure 7, Figure 8 and Figure 9), the dielectric response changes in the way one would predict. The Goldstone mode in the SmC* phase is almost suppressed, while all modes in the SmC_A_* phase change slightly. In Figure 7, the real part of permittivity $\varepsilon_{⊥}^{\prime }$ for several measuring frequencies is shown versus temperature. One can see that the strengths of the P_L_ and P_H_ modes increase under the DC field (compare Figure 4 and Figure 7).

Figure 8 and Figure 9 show the imaginary part $\varepsilon_{⊥}^{\prime \prime }$ of permittivity for several temperatures in both the SmC* and SmC_A_* phases (Figure 8) and in the SmC_A_* phase (Figure 9). In Figure 8, the residual Goldstone mode (G) is seen in the 97–90 °C temperature range. The P_L_ and P_H_ modes start to be detectable at 99 °C and 96 °C, respectively. Again, those modes typical for the SmC_A_* phase coexist with the Goldstone mode typical for the SmC* phase. Please notice that for the temperature range 94–97 °C, there is a clear jump in the strength of the P_L_ mode, while for the 94–96 °C range, there is a clear jump in the strength of the P_H_ mode. Figure 9 shows the results for the SmC_A_* phase. Only the curve for 100 °C does not show any evidence of the P_L_ or P_H_ modes. All three modes (P_L_, P_H_, and S) are well-separated in the frequency domain. At 60 °C, the S mode relaxation frequency is higher than 10 MHz, the P_H_ mode is around 355 kHz, and the P_L_ mode is around 630 Hz. When we compare Figure 6 and Figure 9, it is seen that the P_H_ and P_L_ modes are a little stronger under the DC field while the S mode is a little suppressed. This effect was reported in many papers [20,21,22,29,38,39,40,41]. When the DC field is applied, the ion contribution (at low frequencies) is reduced, and one can see that the amplitude of the P_L_ mode increases when the temperature decreases (it is not clear in Figure 6). Additionally, in the SmC_A_* phase under the DC field, the residual Goldstone mode is observed at frequencies below the P_L_ mode (for temperatures of 95–80 °C).

### 3.3. Results of the Measurements Performed for the Racemic Mixture

**Figure 10:**
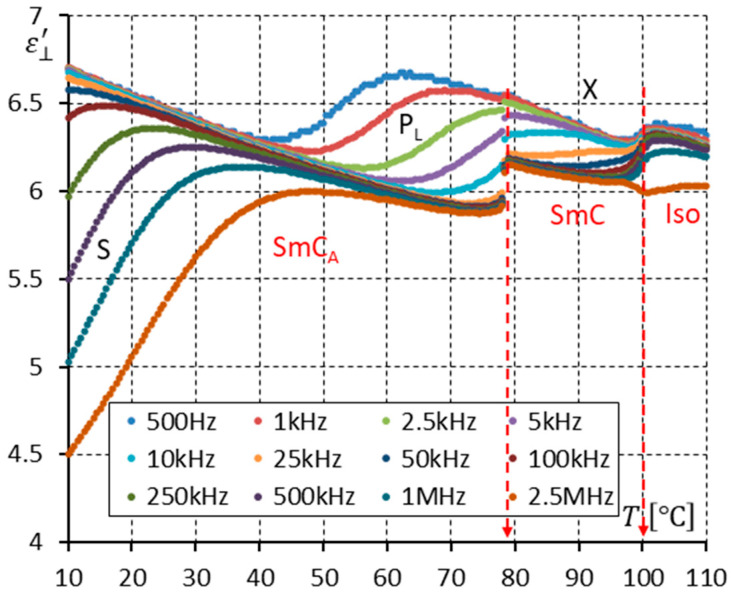
The real part $\varepsilon_{⊥}^{\prime }$ of permittivity for twelve measuring frequencies versus temperature T for the racemate (no DC field applied). Two modes (P_L_, S) are seen in the SmC_A_ phase, and the X mode is observed in the SmC phase. Arrows indicate the phase transitions.

After measuring the enantiomer, measurements of the racemic mixture were performed. Figure 10 shows the real part $\varepsilon_{⊥}^{\prime }$ of permittivity versus temperature for twelve frequencies. Only two modes in the SmC_A_ phase are observed, which can be interpreted as the P_L_ and S modes. The racemate does not exhibit the third mode observed in the SmC_A_* phase—P_H_. The mode visible in the racemic SmC phase, denoted as “X”, seems to continue the P_L_ mode observed in the SmC_A_ phase. The dispersion of the X mode is weaker than that of the P_L_ mode.

**Figure 11:**
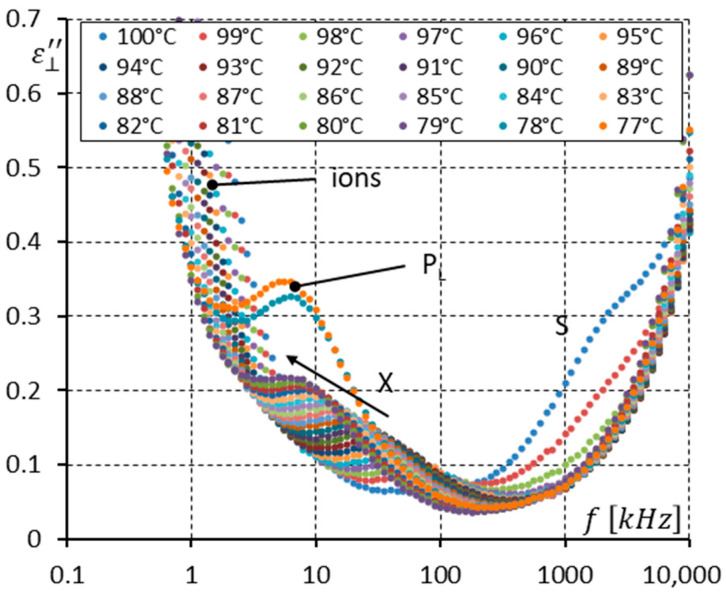
The imaginary part $\varepsilon_{⊥}^{\prime \prime }$ of permittivity for twenty-four temperatures (in the isotropic liquid close to the Iso-SmC phase transition, the SmC phase, and the SmC_A_ phase close to the SmC-SmC_A_ phase transition) versus measuring frequency f for the racemate (no DC field applied). Arrows show how the maximum amplitude of $\varepsilon_{⊥}^{\prime \prime }$ (also the relaxation frequency) shifts when the temperature decreases. Ions are seen at low frequencies.

**Figure 12:**
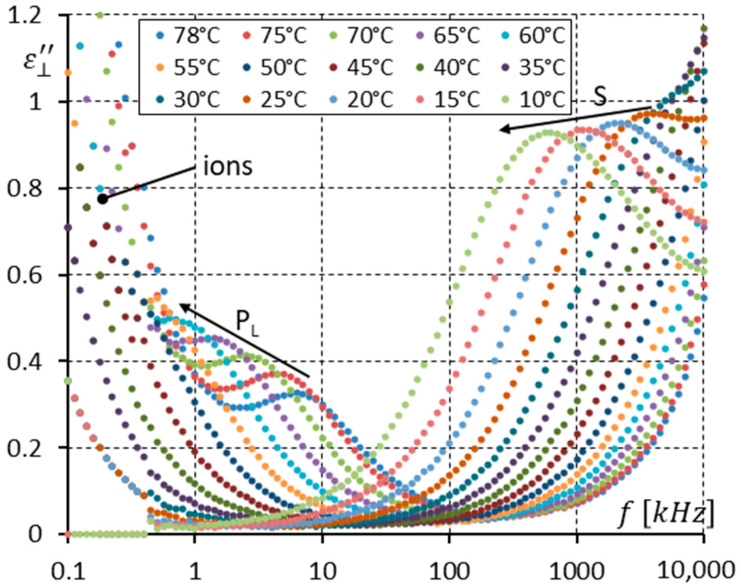
The imaginary part $\varepsilon_{⊥}^{\prime \prime }$ of permittivity for fifteen temperatures (in the SmC_A_ phase) versus measuring frequency f for the racemate (no DC field applied). Arrows show how the maximum amplitude of $\varepsilon_{⊥}^{\prime \prime }$ (also the relaxation frequencies) shifts when the temperature decreases. Ions are seen at low frequencies.

To analyze the dielectric response more precisely, Figure 11 and Figure 12 were prepared. Figure 11 shows the imaginary part $\varepsilon_{⊥}^{\prime \prime }$ of permittivity versus frequency for several temperatures (from 100 to 77 °C) at the Iso-SmC phase transition (100, 99 °C) and in the SmC phase and the SmC_A_ phase (78 and 77 °C). For three temperatures, i.e., 100, 99, and 98 °C, high-frequency relaxation can be interpreted as the molecular motions around the short molecular axis (S mode). The clearing temperature is around 99 °C. This means the S mode shown in Figure 11 corresponds to molecular motion in an isotropic liquid. For temperatures of 97–79 °C, the SmC phase exists, and only one mode is seen. It is denoted as the X mode because it is the first time this mode is analyzed in the synclinic racemic phase. In the SmC phase, the S mode seems to be invisible. When the SmC transforms (while cooling) into the SmC_A_ phase, the X mode does not disappear. Moreover, its strength is doubled. The low-frequency limit of this mode is constant at the temperature of phase transition, while the high-frequency limit is reduced clearly (Figure 10) when entering the SmC_A_ phase.

Figure 12 presents the imaginary part $\varepsilon_{⊥}^{\prime \prime }$ of the dielectric response only in the SmC_A_ phase (temperatures of 78–10 °C). Two modes are seen (P_L_ and S). Please note that the strength of the S mode without a DC field applied in the enantiomer (Figure 6) and racemate (Figure 12) for low temperatures of ~20 °C is practically the same. This is unsurprising because the molecular motions around the short molecular axis should not depend on chirality.

**Figure 13:**
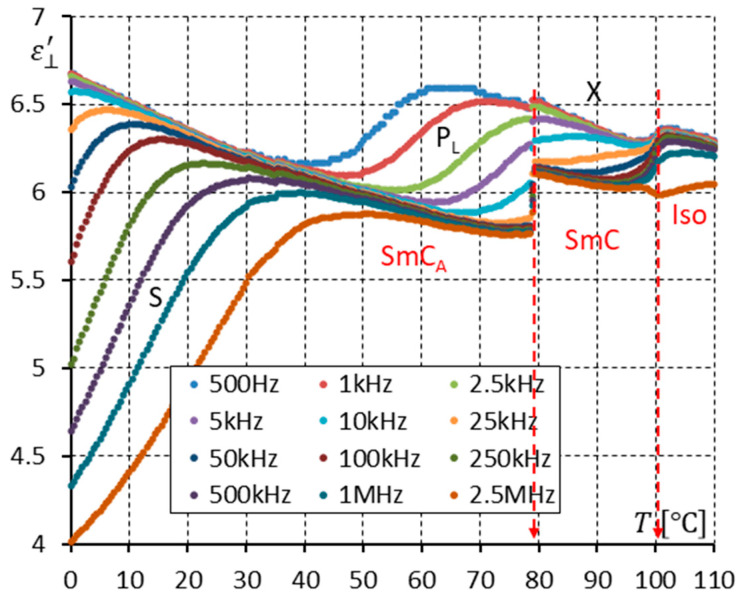
The real part $\varepsilon_{⊥}^{\prime }$ of permittivity for twelve measuring frequencies versus temperature T for the racemate (10 V DC field applied). Two modes (P_L_, S) are seen in the SmC_A_ phase, while the X mode is observed in the SmC phase. Arrows indicate the phase transitions.

**Figure 14:**
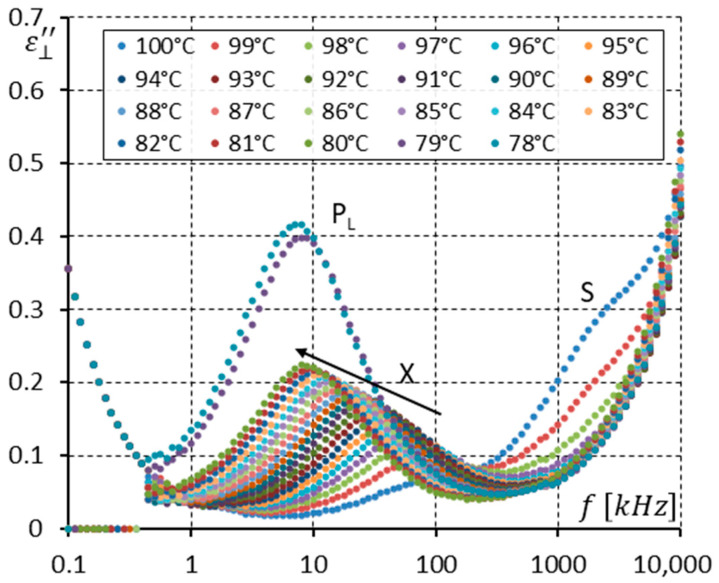
The imaginary part $\varepsilon_{⊥}^{\prime \prime }$ of permittivity for twenty-three temperatures (in the isotropic liquid close to the Iso-SmC phase transition, the SmC, and the SmC_A_ close to the SmC-SmC_A_ phase transition) versus measuring frequency f for the racemate (10 V DC field applied). The arrow shows how the maximum amplitude of $\varepsilon_{⊥}^{\prime \prime }$ (also the relaxation frequency) shifts when the temperature decreases.

**Figure 15:**
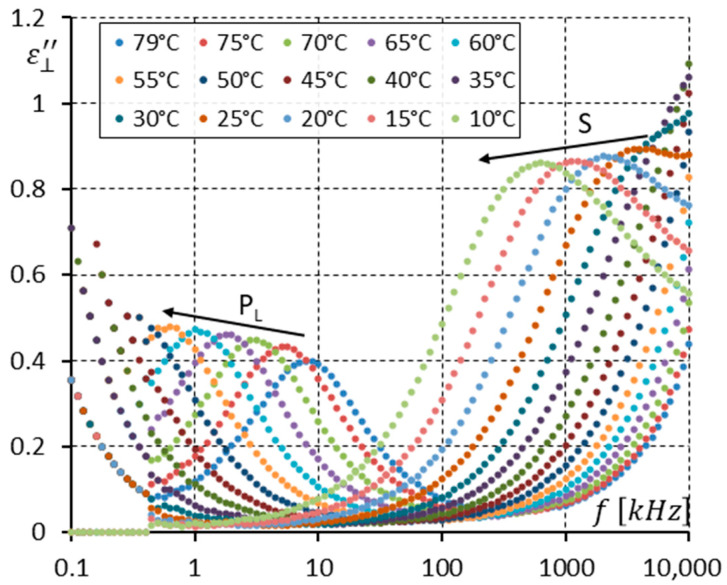
The imaginary part $\varepsilon_{⊥}^{\prime \prime }$ of permittivity for fifteen temperatures (in the SmC_A_ phase) versus measuring frequency f for the racemate (10 V DC field applied). Arrows show how the maximum amplitude of $\varepsilon_{⊥}^{\prime \prime }$ (also the relaxation frequencies) shifts when the temperature decreases.

One can see in Figure 11 and Figure 12 that the imaginary part $\varepsilon_{⊥}^{\prime \prime }$ of permittivity at low frequency is influenced by the presence of ions. The DC field (10 V) was applied to suppress the ion contribution to the dielectric response. Additionally, it helped to observe how the DC field modifies the dielectric response in the racemate. The results are presented in Figure 13, Figure 14 and Figure 15.

Figure 10 and Figure 13 show the real part $\varepsilon_{⊥}^{\prime }$ of permittivity. They seem to be very similar. One can only find that the high-frequency limits of permittivity in the SmC_A_ and SmC phases close to the SmC-SmC_A_ phase transition are lower for permittivity measured with the DC field (Figure 13) than without the DC field (Figure 10).

Essential differences can be seen when comparing Figure 11 and Figure 14, where the imaginary parts $\varepsilon_{⊥}^{\prime \prime }$ of permittivity in the Iso, SmC, and SmC_A_ phases are presented (without and with the DC field, respectively). Because of the effective suppression of the ion contribution, the imaginary part $\varepsilon_{⊥}^{\prime \prime }$ of permittivity is well visible in Figure 14 at low frequencies. One can notice that the P_L_ mode is strengthened under a DC field. Similarly, the X mode in the SmC phase is strengthened under a DC field. The X mode behaves like the P_L_ mode in the SmC_A_ phase.

When comparing Figure 12 and Figure 15, where the imaginary part $\varepsilon_{⊥}^{\prime \prime }$ of permittivity in the SmC_A_ phase is presented (without and with the DC field, respectively), the P_L_ mode is stronger under the DC field, while the S mode is weaker under the DC field.

Three-dimensional plots of the imaginary part $\varepsilon_{⊥}^{\prime \prime }$ of permittivity versus frequency and temperature are presented in the Supplementary Materials.

### 3.4. Results of the Calculations

**Figure 16:**
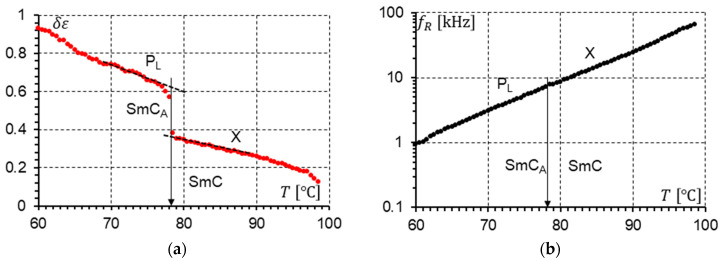
(**a**) The strengths δε of the P_L_ and X modes versus temperature at the SmC_A_-SmC phase transition in the racemate. No DC field was applied. The phase transition is marked by an arrow. (**b**) The relaxation frequency $f_{R}$ of the P_L_ and X modes versus temperature at the SmC_A_-SmC phase transition in the racemate. No DC field was applied. The phase transition is marked by an arrow. The dashed line in the $\delta \varepsilon \left(T\right)$ plot is the linear approximation of $\delta \varepsilon \left(T\right)$ near the phase transition.

**Figure 17:**
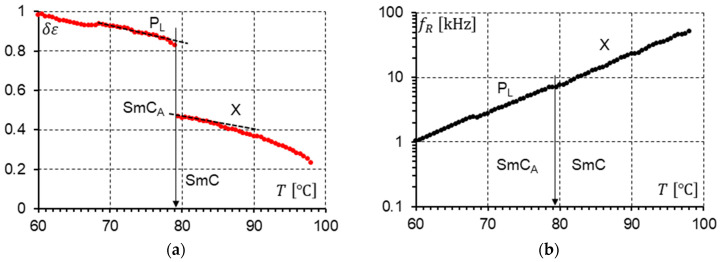
(**a**) The strengths δε of the P_L_ and X modes versus temperature at the SmC_A_-SmC phase transition in the racemate. A 10 V DC field was applied. The phase transition is marked by an arrow. (**b**) The relaxation frequency $f_{R}$ of the P_L_ and X modes versus temperature at the SmC_A_-SmC phase transition in the racemate. A 10 V DC field was applied. The phase transition is marked by an arrow. The dashed line in the $\delta \varepsilon \left(T\right)$ plot is the linear approximation of $\delta \varepsilon \left(T\right)$ near the phase transition.

The parameters describing the modes presented in the dielectric response were determined to fit calculated permittivity using Formula (1) with the experimental results. For the racemate, the results of the calculations for temperatures close to the SmC-SmC_A_ phase transition are presented in Figure 16 (measurements without a DC field) and Figure 17 (measurements under a 10 V DC field). Both figures present the dielectric strengths of the detected modes (P_L_ and X) and relaxation frequencies of the detected modes (P_L_ and X). One can notice that the phase transition SmC-SmC_A_ is seen only in Figure 16a and Figure 17a. The dielectric strength plot is not continuous at the SmC-SmC_A_ phase transition.

When the plot of the relaxation frequency $f_{R}$ versus temperature T (Figure 16b and Figure 17b) is considered, no phase transition can be seen. The relaxation frequency changes continuously. When the DC field is on, the dielectric strength of the P_L_ and X modes increases. The X mode shows a similar behavior as the P_L_ mode. It is worth highlighting that at cooling, when the SmC phase transforms into the SmC_A_ phase, the dielectric strength increases almost twice (0.63/0.36 = 1.75—without DC field; 0.85/0.47 = 1.81—with 10 V DC field). This value would probably be closer to two when the structure is perfect. This is probably related to the fact that in the synclinic SmC phase, the unit structure consists of one layer, while in the anticlinic SmC_A_ phase, it consists of two layers. Hence, a doubled structure gives doubled strength.

**Figure 18:**
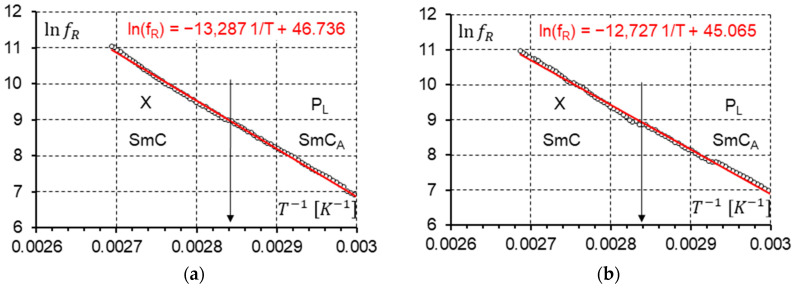
The Arrhenius plot for the P_L_ and X modes at the SmC_A_-SmC phase transition in the racemate. The phase transition is marked by an arrow. (**a**) No DC field is applied. (**b**) A 10 V DC field is applied. The red line is the linear approximation of $\ln f_{R}\left(T^{-1}\right)$.

Knowing the temperature dependence of the relaxation frequencies of the X and P_L_ modes shown in Figure 16b and Figure 17b, we can use the Arrhenius law (Equation (2)) to determine the activation energy $E_{a}$. This is possible when the observed modes fulfill the Arrhenius model. Finally, the activation energy $E_{a}$ was calculated. The plots prepared for the measurements without (Figure 18a) and with the 10 V DC field (Figure 18b) give almost the same results for activation energy as follows: $E_{a}$ = 1.14 eV/molecule (Figure 18a) and $E_{a}$ = 1.10 eV/molecule (Figure 18b). The activation energy is the same for both modes (P_L_ and X).

When the similarities between the P_L_ (in the SmC_A_ phase) and X (in the SmC phase) modes are shown, we can calculate the parameters of the modes observed in the racemic SmC_A_ and the enantiomeric SmC_A_*. Below, we show only the results obtained for racemate and enantiomer under the influence of the 10 V DC field because the results measured without a DC field are very similar. Only amplitudes of observed modes in the anticlinic phases (SmC_A_ and SmC_A_*) change under the DC field. Additionally, the DC field reduces the ion contribution to the dielectric response, making it easier to find the parameters of the P_L_ mode.

Firstly, it must be mentioned that the quality of the relaxation parameters determination depends on the measuring range of the used equipment. In our case, the parameters of the P_L_ mode can be well determined if its relaxation frequency is higher than 200 Hz, while the parameters of the S mode can be well determined if its relaxation frequency is lower than 10 MHz. This is guaranteed by the method presented in [35]. The parameters of the P_H_ mode are calculated more precisely because its relaxation frequency is in the middle of the measuring range of our impedance analyzer.

**Figure 19:**
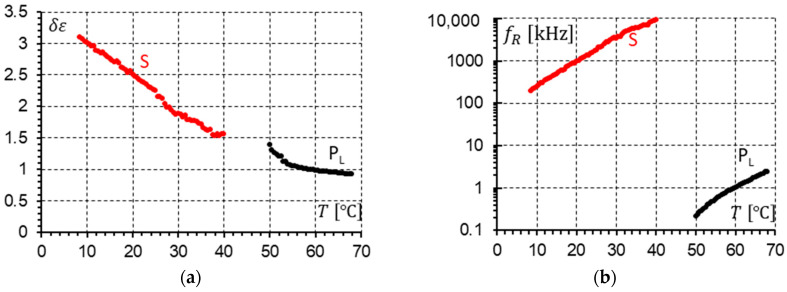
(**a**) The strengths δε of the P_L_ and S modes versus temperature in the SmC_A_ phase in the racemate. (**b**) The relaxation frequency $f_{R}$ of the P_L_ and S modes versus temperature in the SmC_A_ phase transition in the racemate. A 10 V DC field was applied.

**Figure 20:**
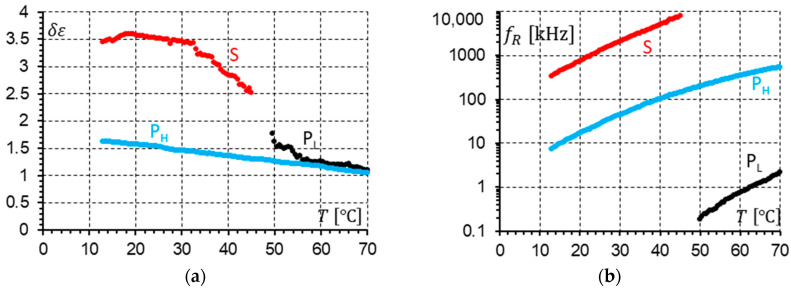
(**a**) The strengths δε of the P_L_, P_H_, and S modes versus temperature in the SmC_A_* phase in the enantiomer. (**b**) The relaxation frequency $f_{R}$ of the P_L_, P_H_, and S modes versus temperature in the SmC_A_* phase in the enantiomer. A 10 V DC field was applied.

When comparing Figure 19b and Figure 20b, it is seen that the relaxation frequencies of the S and P_L_ modes in the SmC_A_ (racemate) and SmC_A_* (enantiomer) phases are the same for measurements performed with a 10 V DC field. In SmC_A_*, additionally, the P_H_ mode is observed with an amplitude similar to the P_L_ mode (Figure 20a). The P_H_ mode coexists with the S and P_L_ modes in different temperature ranges. We cannot simultaneously see (similar to the racemic SmC_A_) the S and P_L_ modes at the same temperature.

## 4. Discussion, Conclusions, and Summary

Previous dielectric studies were limited to pure antiferroelectric and ferroelectric enantiomers. Only three papers were devoted to dielectric studies of the “antiferroelectric” racemates [17,18,19], while the “ferroelectric” racemate did not raise interest so far. The dielectric response of the SmC_A_* phase is well described in many papers [20,21,22,23,24,38,39,40,41,42,43,44]. Four modes in the antiferroelectric (SmC_A_*) phase were found.

Two molecular modes are related to individual motions of molecules around a long molecular axis (L mode) and a short molecular axis (S mode). The L mode’s relaxation frequency is higher than the measuring range of our experimental setup. Hence, only the S mode was detected in the experiment shown here. The sample should be cooled enough (below 30 °C) to make this mode visible in measurements. The S mode and the L mode are Arrhenius-type modes. This means that their relaxation frequencies decrease with a decrease in temperature. To observe the L mode, the measurement temperature should be below −50 °C [45]. In the presented experiment, the lowest temperature was higher than 0 °C. 

The next two modes (P_L_ and P_H_) were described as “non-cancelled phason modes” [20,21,23]. Their amplitude is lower than the amplitude of the S mode. This means that they are weak.

For the same temperature, the relaxation frequency of the P_H_ mode is 300–500 times higher than the relaxation of the P_L_ mode. One may think that the P_L_ mode seems similar to the Goldstone mode in the ferroelectric SmC* phase, but our results show that the dielectric responses of the Goldstone mode and the P_L_ mode at the phase transition exhibit different relaxation frequencies. Our results show that Goldstone and P_L_ modes can coexist at temperatures close to the SmC*-SmC_A_* phase transition. Both the P_H_ and P_L_ modes are stronger under a low DC field—this effect is also presented in this paper. However, when we apply a high enough DC field, the helical structure becomes unwound, and both the P_H_ and P_L_ modes disappear [29]. This means that unwound (by a DC field) helical structures suppress the Goldstone, P_L_, and P_H_ modes. Moreover, this paper confirms that unwinding the helical structure in the SmC_A_* phase is more difficult than in the SmC* phase.

The racemization changes the dielectric response in comparison with the pure enantiomer. One can observe several effects, which can be found using dielectric spectroscopy. Racemization fully suppresses the P_H_ mode in the SmC_A_ phase (Figure 10, Figure 12, Figure 13 and Figure 15). This mode is visible in the enantiomer only (Figure 4, Figure 6, Figure 7 and Figure 9). Simultaneously in the racemate, the P_L_ and S modes in the SmC_A_ phase are still detectable as they are in the enantiomeric SmC_A_* phase (compare Figure 4 and Figure 10 and compare Figure 6 and Figure 12). The P_L_ mode in the racemic SmC_A_ phase is amplified under the DC field (compare Figure 10 and Figure 13 and compare Figure 12 and Figure 15), similar to that in the enantiomeric SmC_A_* phase (compare Figure 4 and Figure 7 and compare Figure 6 and Figure 9). Simultaneously, the S mode in the racemic SmC_A_ phase is attenuated under the DC field (Figure 12 and Figure 15) as it is in the enantiomeric SmC_A_* phase (Figure 6 and Figure 9). The relaxation frequencies of the P_L_ and S modes in the racemic SmC_A_ phase (Figure 19b) are approximately the same as the relaxation frequencies of the P_L_ and S modes in the enantiomeric SmC_A_* phase (Figure 20b). Moreover, the S mode is stronger than the P_L_ mode in the racemic SmC_A_ phase (Figure 19a) and in the enantiomeric SmC_A_* phase (Figure 20a). The X mode found in the racemic SmC phase seems to continue the P_L_ mode from the racemic SmC_A_ phase. Additionally, the amplitude δε of the P_L_ mode in the racemic SmC_A_ phase is almost twice as high as the amplitude of the X mode in the racemic SmC phase close to the SmC-SmC_A_ phase transition (Figure 16a and Figure 17a). A similar effect is visible at the SmC*-SmC_A_* phase transition (Figure 8). The Goldstone mode is suppressed when the 10V DC field is on in the SmC* phase, and weak P_L_ and P_H_ modes are well seen. A similar effect when racemization reveals modes, normally covered by a strong Goldstone mode, was observed earlier [46]. The P_L_ mode is amplified by a factor of two when the SmC* changes into the SmC_A_* phase (compare plots for temperatures of 97 and 95 °C). The relaxation frequency $f_{R}$ of the X mode in the racemic SmC phase decreases with as the temperature decreases, and the same is observed for the relaxation frequency of the P_L_ mode in the racemic SmC_A_ (Figure 16b and Figure 17b). One can see the continuous evolution of the relaxation frequency at the SmC-SmC_A_ phase transition. The X mode in the racemic SmC phase is amplified under the DC field as it is for the P_L_ mode in the racemic SmC_A_ phase (Figure 11 and Figure 14). One should remember that ions can distort the low-frequency imaginary part of complex permittivity—the DC field suppresses this effect efficiently. The Goldstone mode is not detectable in the racemic SmC phase (compare Figure 4 and Figure 10 and compare Figure 5 and Figure 11). The Goldstone mode in the enantiomeric SmC* phase exhibits a lower relaxation frequency than the P_L_ mode in the enantiomeric SmC_A_* phase (Figure 5 and Figure 8). These two modes are different—they have nothing to do with each other.

One should remember that molecules in the racemate are still chiral. But the equimolar R- and S-enantiomer ratio makes the mixture optically inactive (the helicoidal structure disappears). Racemization is the procedure that should suppress or eliminate all material properties related to chirality. This is obviously seen in the disappearance of the Goldstone mode in the racemic SmC phase. It is still unclear why only the P_H_ mode is suppressed in SmC_A_ by racemization while the P_L_ mode seems to be not influenced by racemization; a theoretical explanation is required. This experimental fact should change the opinion that the P_L_ and P_H_ modes have similar origins in chirality [20,21,38,39]. The fact that both the P_L_ and P_H_ modes exhibit very similar behavior in the enantiomer does not rule out the possibility that both modes will behave completely differently in the racemate. Moreover, the racemization in the racemic SmC phase reveals the X mode, similar to the P_L_ mode in the racemic SmC_A_ and the enantiomeric SmC_A_* phases. A similar continuous transformation of the mode observed in the SmC and SmC_A_ phases was observed in an achiral compound [47]. When racemization eliminates the strong Goldstone mode, a weak X mode (P_L_ mode-like) can be visible. When we compare the results from Figure 8 and Figure 14, we can venture to say that the P_L_ and P_H_ modes start to be seen in the SmC* phase (close to the SmC*-SmC_A_* phase transition) when the Goldstone mode is suppressed by the DC field. Similarly, the residual Goldstone mode is still detected in the SmC_A_* phase (close to the SmC*-SmC_A_* phase transition).

The conclusions about dielectric modes are collected in Table 1 (for enantiomer) and Table 2 (for racemate).

## Figures and Tables

**Table 1:** Dielectric modes observed in enantiomeric anticlinic SmC_A_* and synclinic SmC* phases when the DC field is off and on.

	SmC_A_*	SmC*
Dielectric modes (no DC)	Residual Goldstone mode P_L_ mode P_H_ mode Molecular S mode	Goldstone mode Weak P_L_ mode covered by the Goldstone mode Weak P_H_ mode covered by the Goldstone mode Molecular S mode
Dielectric modes (with DC)	Residual Goldstone mode P_L_ mode strengthened by the DC field P_H_ mode strengthened by the DC field Molecular S mode weakened by the DC field	Suppressed Goldstone mode P_L_ mode strengthened by the DC field, non-covered by the suppressed Goldstone mode P_H_ mode strengthened by the DC field, non-covered by the suppressed Goldstone mode Molecular S mode weakened by the DC field

**Table 2:** Dielectric modes observed in racemic anticlinic SmC_A_ and synclinic SmC phases when the DC field is off and on.

	SmC_A_	SmC
Dielectric modes (no DC)	P_L_ mode Molecular S mode	Weak X(P_L_) mode Molecular S mode
Dielectric modes (with DC)	P_L_ mode strengthened by the DC field Molecular S mode weakened by the DC field P_L_ mode strengthened by the DC field P_H_ mode strengthened by the DC field Molecular S mode weakened by the DC field	X(P_L_) mode strengthened by the DC field Molecular S mode weakened by the DC field P_H_ mode strengthened by the DC field, non-covered by the suppressed Goldstone mode Molecular S mode weakened by the DC field

## Data Availability

The data presented in this study are available on request from the corresponding author (due to privacy).

## Supplementary Materials

The following supporting information can be downloaded at: https://www.mdpi.com/article/10.3390/ma17133335/s1. Figure S1. DSC plot for enantiomer. Figure S2. DSC plot for racemate. Figure S3. 3D plot of the imaginary part *ε*_⊥_″ of permittivity versus temperature *T* [°C] and frequency *f* [kHz] for the enantiomer (10 V DC field applied). The SmC* phase under 10 V DC field does not show any relaxation. Figure S4. 3D plot of the imaginary part *ε*_⊥_″ of permittivity versus temperature *T* [°C] and frequency *f* [kHz] for the racemate (10 V DC field applied). Figure S5. 3D plot of the imaginary part *ε*_⊥_″ of permittivity versus temperature *T* [°C] and frequency *f* [kHz] for the enantiomer (5 V DC field applied). The SmC* phase under 5 V DC field shows residual Goldstone mode. Figure S6. 3D plot of the imaginary part *ε*_⊥_″ of permittivity versus temperature *T* [°C] and frequency *f* [kHz] for the racemate (5 V DC field applied). Figure S7. 3D plot of the imaginary part *ε*_⊥_″ of permittivity versus temperature *T* [°C] and frequency *f* [kHz] for the enantiomer (no DC field applied). The SmC* phase without the DC field shows strong Goldstone mode. Figure S8. 3D plot of the imaginary part *ε*_⊥_″ of permittivity versus temperature *T* [°C] and frequency *f* [kHz] for the enantiomer (no DC field applied). The SmC* phase without the DC field shows strong Goldstone mode. Figure S9. 3D plot of the imaginary part *ε*_⊥_″ of permittivity versus temperature *T* [°C] and frequency *f* [kHz] for the racemate (no DC field applied).
