# Supplementary material for: Dielectric Modes in Antiferroelectric and Ferroelectric Liquid Crystals in a Pure Enantiomeric Version and a Racemic Mixture
Source: Materials (Basel). 2024 Jul 5;17(13):3335. doi: 10.3390/ma17133335 (PMC11243658; doi:10.3390/ma17133335)
Supplement: Supplementary file 1 [file materials-17-03335-s001.zip › materials-3040111-supplementary.pdf]

Supplementary file for the paper entitled: *Dielectric modes in antiferroelectric and ferroelectric liquid crystal in pure enantiomeric version and racemic mixture* published in Materials 2024.

P. Perkowski[1], M. Urbańska[2]

[1]*Institute of Applied Physics* [2]*Institute of Chemistry*
*Military University of Technology, Warsaw, POLAND*

This supplemental file presents:

1. The DSC plots for enantiomer and racemate.

2. 3D plots: the imaginary part of permittivity *vs*. frequency and temperature. 3D plots are prepared to present electric properties comprehensively.

## 1. DSC plots

Phase transition temperatures and enthalpies were determined by a differential scanning calorimeter using the DSC „SETARAM" 141 instruments with a scanning rate of 2°C/min during heating and cooling cycles in a nitrogen atmosphere. The weight of each sample was about 20-30 mg. Two types of phase transition sequences are observed: Cr-SmC$_A$*-SmC*-SmA*-Iso and Cr-SmC$_A$-SmC-Iso. DSC diagrams for enantiomer and racemate are respectively presented in Figures S1 and S2.

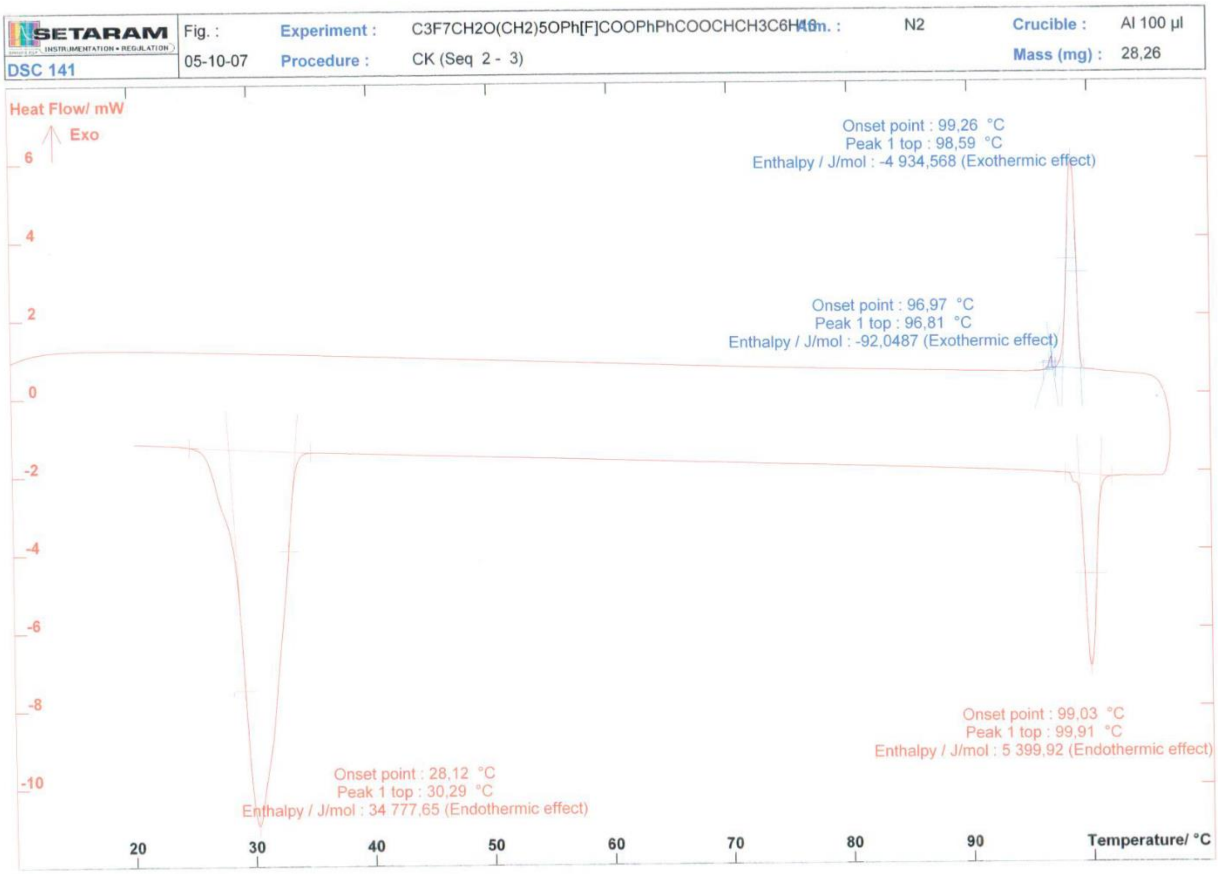


Figure S1. DSC plot for enantiomer.

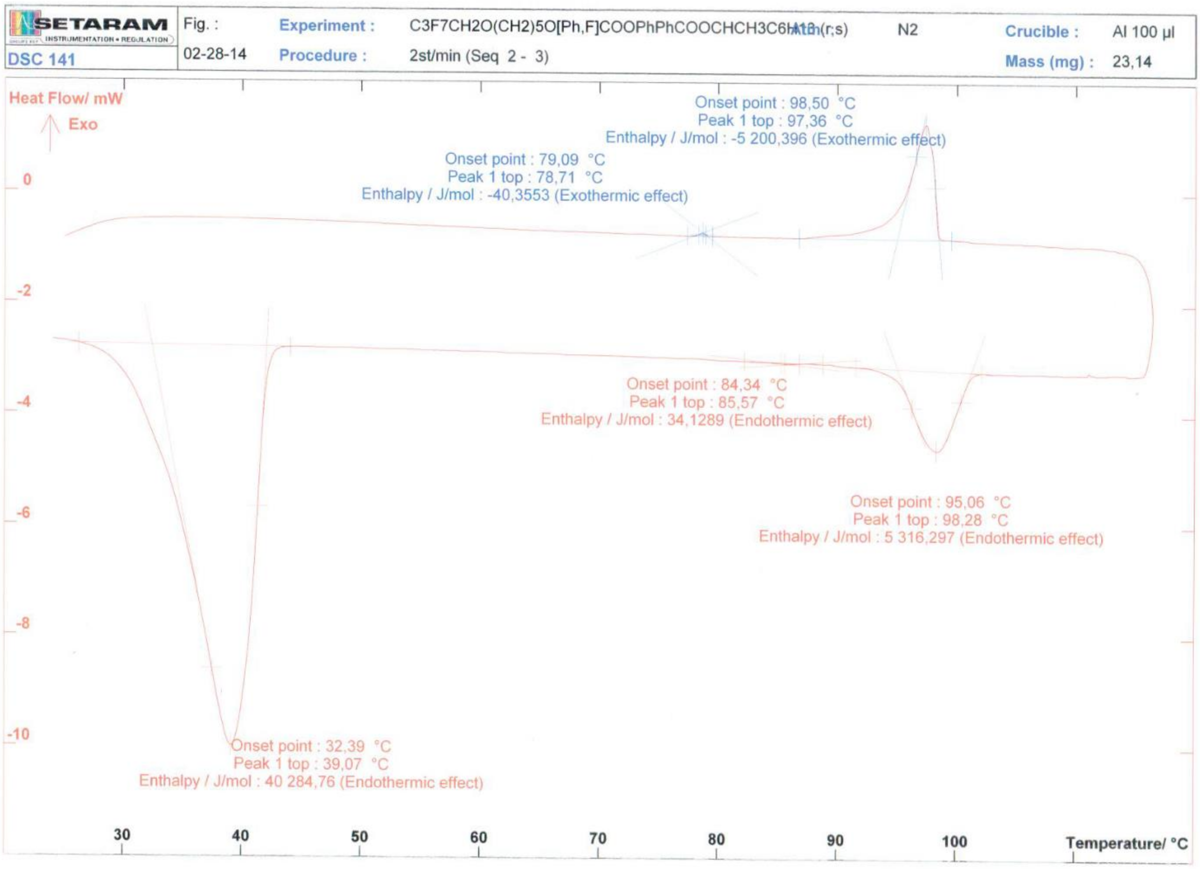


Figure S2. DSC plot for racemate.

## 2. 3D plots

We present 3D plots (Figures S3-S9) of the imaginary part $\varepsilon_{\perp}''$ of permittivity versus the temperature $T$ [°C] and the frequency $f$ [kHz] for enantiomer (Figures S3, S5, S7, S8) and for racemate (Figures S4, S6, S9). Detected modes (G, $P_L$, $P_H$, S and X) are marked on the figures. To suppress the Goldstone mode and to strengthen $P_L$ and $P_H$ modes, we applied 5 V or 10 V DC fields.

One can see that $P_H$-mode, seen in the $SmC_A$* phase (Figures S3, S5, S7, S8), does not exist in the $SmC_A$ phase (Figures S4, S6, S9). Additionally, it is seen that the Goldstone mode, presented in the SmC* (Figures S5, S7, S8), disappears in the SmC phase (S4, S6, S9) and is suppressed by the 10V DC field (Figure S3), and the X-mode presented in the SmC phase is the reduced continuation of $P_L$-mode from the $SmC_A$ phase (Figures S4, S6, S9). The S-mode (the molecular motion around the short molecular axis is detectable both in $SmC_A$* / $SmC_A$ and in the isotropic liquid (Figures S3-S9).

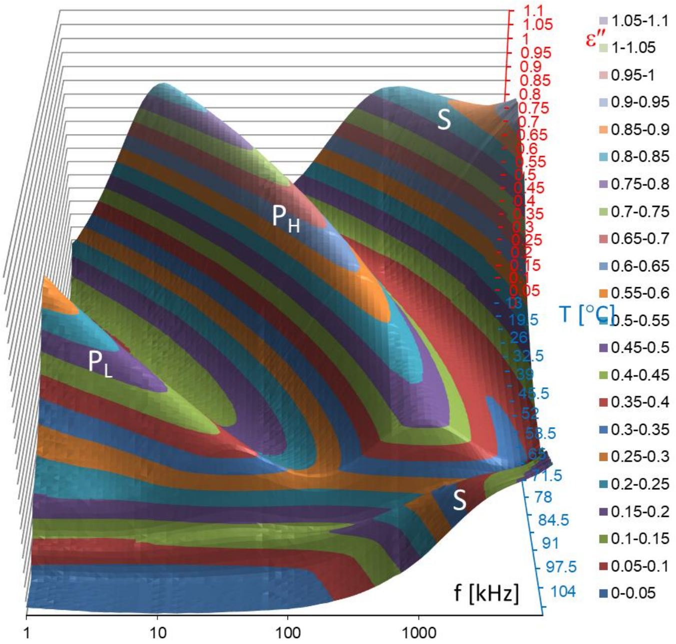


Figure S3. 3D plot of the imaginary part $\varepsilon''_{\perp}$ of permittivity versus temperature $T$ [℃] and frequency $f$ [kHz] for the enantiomer (10 V DC field applied). The SmC* phase under 10 V DC field does not show any relaxation.

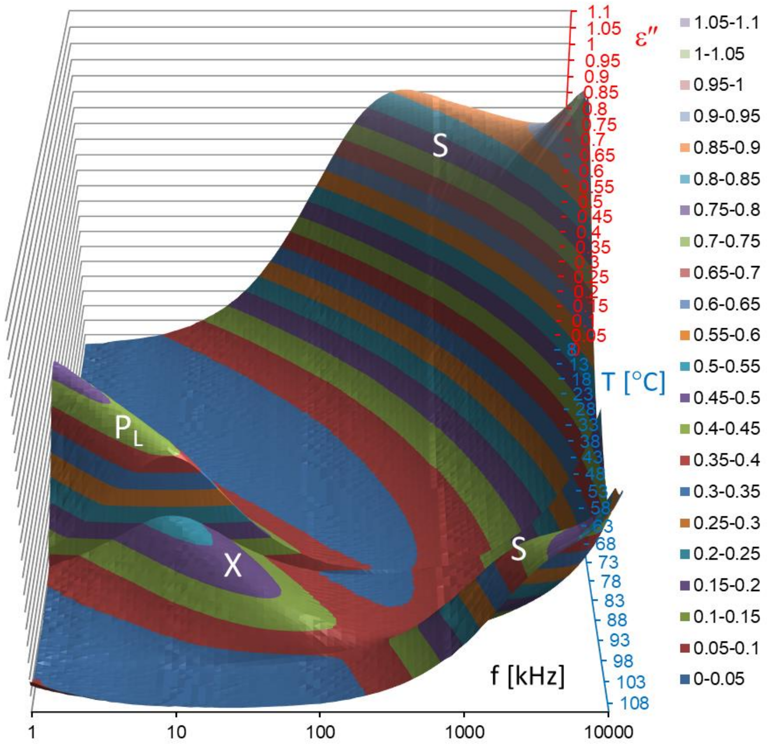


Figure S4. 3D plot of the imaginary part $\varepsilon''_{\perp}$ of permittivity versus temperature $T$ [℃] and frequency $f$ [kHz] for the racemate (10 V DC field applied).

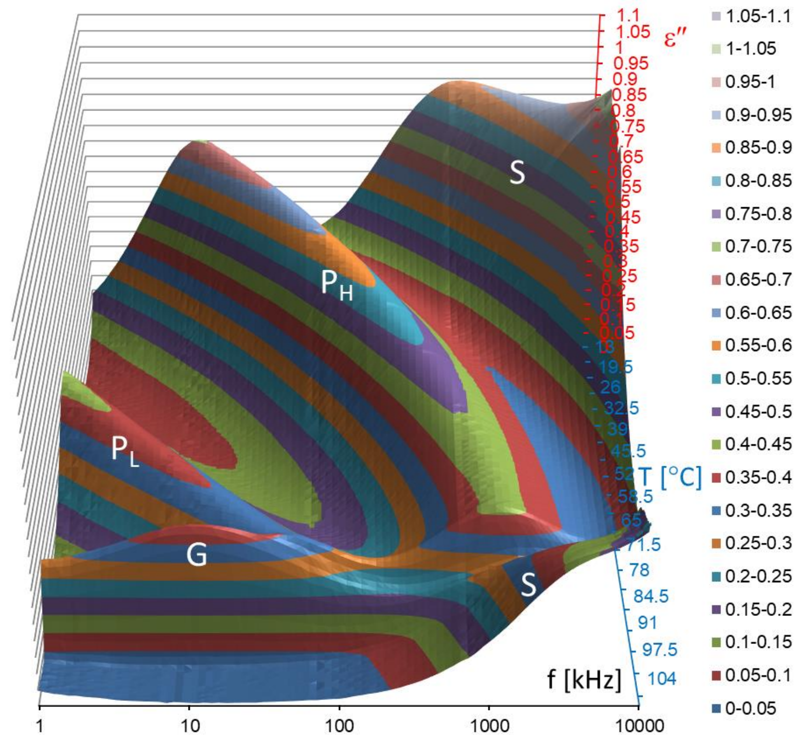


Figure S5. 3D plot of the imaginary part $\varepsilon''_{\perp}$ of permittivity versus temperature $T$ [℃] and frequency $f$ [kHz] for the enantiomer (5 V DC field applied). The SmC* phase under 5 V DC field shows residual Goldstone mode.

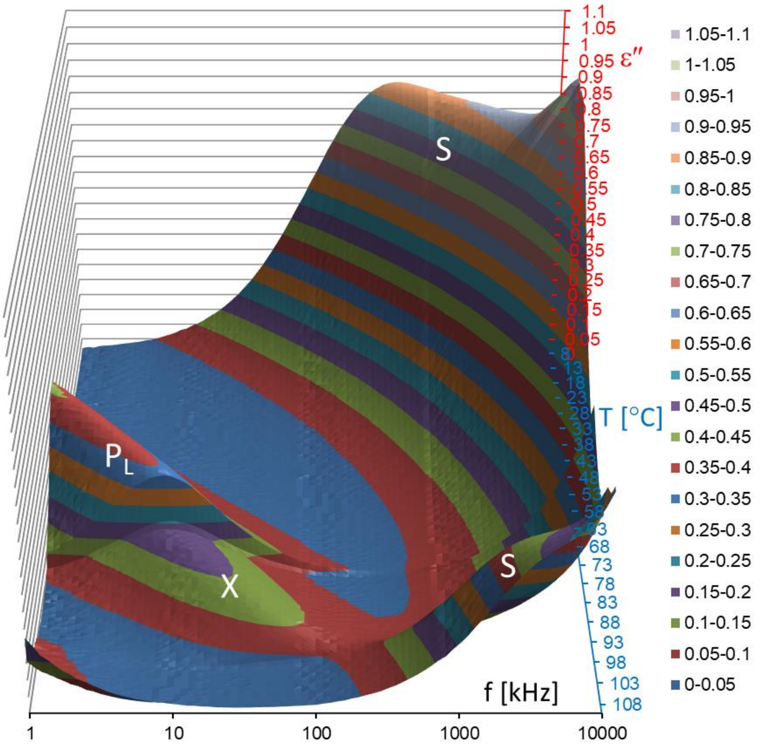


Figure S6. 3D plot of the imaginary part $\varepsilon''_{\perp}$ of permittivity versus temperature $T$ [℃] and frequency $f$ [kHz] for the racemate (5 V DC field applied).

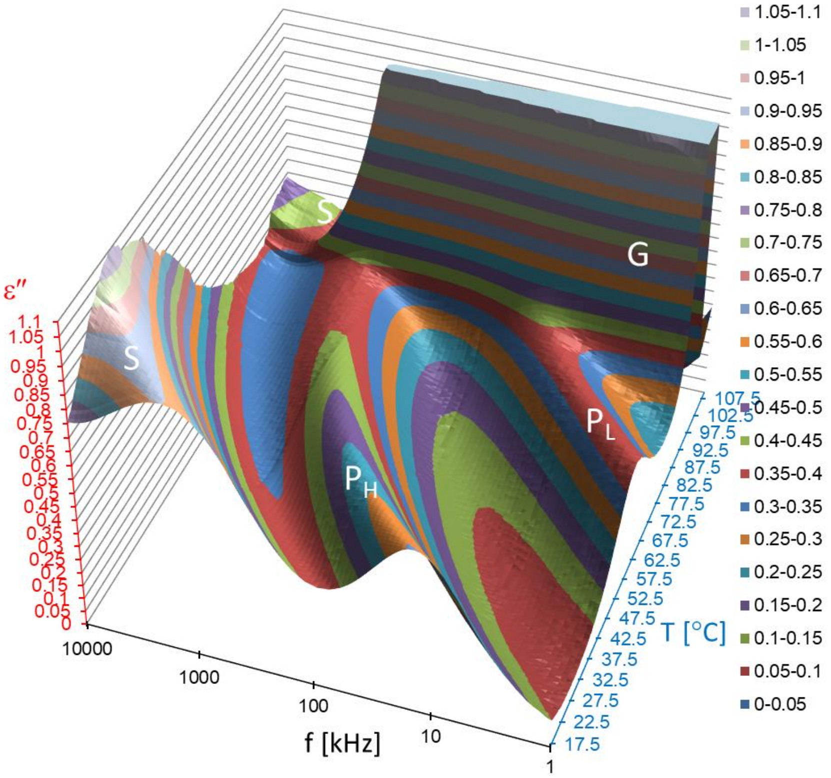


Figure S7. 3D plot of the imaginary part $\varepsilon_{\perp}''$ of permittivity versus temperature $T\,[℃]$ and frequency $f\,[\mathrm{kHz}]$ for the enantiomer (no DC field applied). The SmC* phase without the DC field shows strong Goldstone mode.

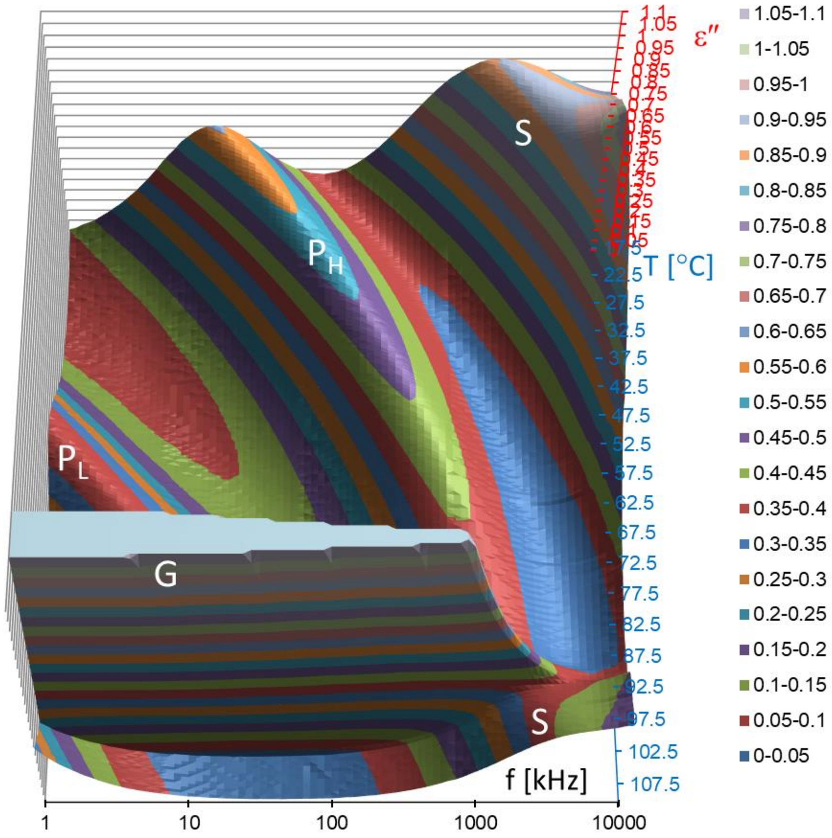


Figure S8. 3D plot of the imaginary part $\varepsilon_{\perp}''$ of permittivity versus temperature $T\,[℃]$ and frequency $f\,[\mathrm{kHz}]$ for the enantiomer (no DC field applied). The SmC* phase without the DC field shows strong Goldstone mode.

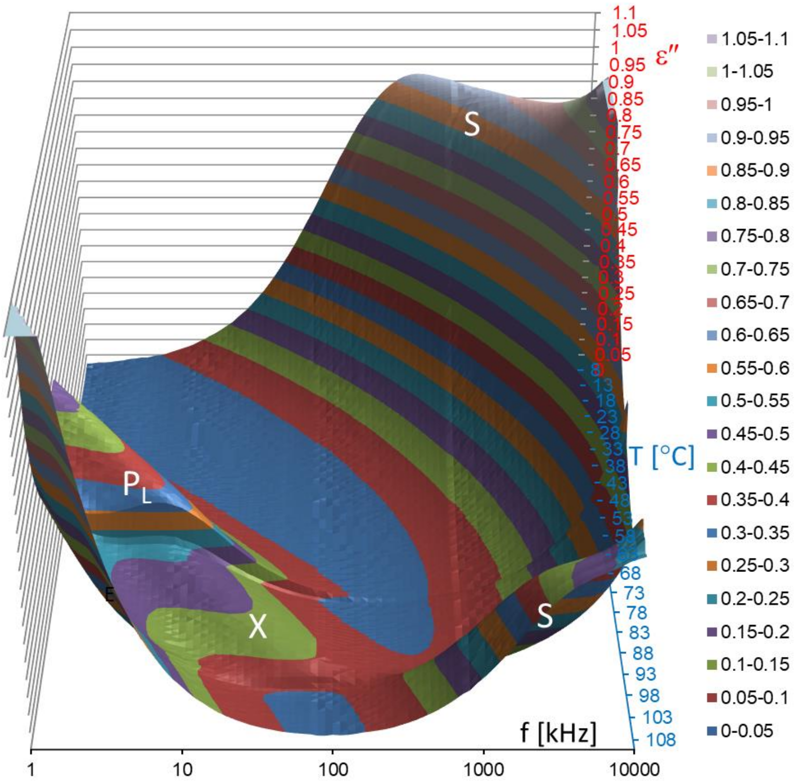


Figure S9. 3D plot of the imaginary part $\varepsilon_{\perp}''$ of permittivity versus temperature $T$ [℃] and frequency $f$ [kHz] for the racemate (no DC field applied).
